# Fertility Desire and Associations with Condomless Sex, Antiretroviral Adherence, and Transmission Potential in a Cohort of Kenyan Women Living with HIV in Sero-discordant Relationships: A Mixed Methods Study

**DOI:** 10.1007/s10461-023-04004-4

**Published:** 2023-02-09

**Authors:** McKenna C. Eastment, John Kinuthia, Kenneth Tapia, George Wanje, Katherine Wilson, Anne Kaggiah, Jane M. Simoni, Kishorchandra Mandaliya, Danielle N. Poole, Barbra A. Richardson, Walter Jaoko, Grace John-Stewart, R. Scott McClelland

**Affiliations:** 1 Department of Medicine, University of Washington, 325 9th Ave, Box 359909, Seattle, WA 98104 USA; 2 Department of Global Health, University of Washington, Seattle, WA USA; 3 Kenyatta National Hospital, Nairobi, Kenya; 4 Department of Medical Microbiology and Immunology, University of Nairobi, Nairobi, Kenya; 5 Department of Psychology, University of Washington, Seattle, WA USA; 6 Department of Geography, Dartmouth College, Hanover, NH USA; 7 Department of Biostatistics, University of Washington, Seattle, WA USA; 8 Department of Epidemiology, University of Washington, Seattle, WA USA; 9 Department of Pediatrics, University of Washington, Seattle, WA USA

**Keywords:** Fertility desire, Fertility intent, Women living with HIV, Sero-discordant partnerships

## Abstract

For women living with HIV (WLH) in serodiscordant partnerships, decisions about childbearing can challenge condom use and antiretroviral adherence. In a prospective cohort of 148 WLH in serodiscordant partnerships, 58 (39%) wanted more children in the future but were not currently trying to conceive (fertility desire), and 32 (22%) were currently trying to become pregnant (fertility intent). Detection of prostate specific antigen (PSA) in vaginal secretions, a marker for recent condomless sex, was lowest in women with fertility desire and highest in women with fertility intent. Detectable viral load followed a similar pattern. Risk of HIV transmission, when condomless sex and PSA detection occurred concurrently, was three to fourfold higher at visits with fertility intent compared to visits with fertility desire. Qualitative interviews underscored the importance women place on childbearing and suggested that they had limited information about the role of antiretroviral therapy in reducing sexual HIV transmission.

## Introduction

Sub-Saharan Africa (SSA) has the highest prevalence and incidence of human immunodeficiency virus (HIV) infection worldwide [1]. Nearly 60% of people living with HIV in SSA are women of childbearing age [2]. Estimates suggest between 55.1 and 92.7% of all new HIV infections occur in stable heterosexual serodiscordant partnerships, where one partner has HIV and the other remains seronegative [3, 4]. Women living with HIV (WLH) of childbearing age continue to express desire for children but may face challenges with safe conception particularly in the setting of discordant partnerships [5–8]. Studies from Ethiopia and Zambia found that being in a discordant relationship with a partner who is seronegative was associated with fertility desire [5, 9]. Despite concerns about HIV transmission during pregnancy, people living with HIV in serodiscordant partnerships desire future children and must navigate reproduction with the added consideration of HIV transmission [10, 11].

While there are available methods for safer conception including use of antiretroviral therapy (ART) for the partner living with HIV and pre-exposure prophylaxis (PrEP) for the partner not living with HIV [12], discordant couples may face challenges of unplanned condomless sex, unsuppressed viral loads, and HIV transmission in the context of reproduction. Special counseling may be warranted for WLH about their reproductive health in the context of fertility desire and fertility intent [13]. A study in 2017 documented that use of both modern contraceptives and condoms were rare in discordant couples in Africa, raising the possibility of unintended pregnancy and HIV transmission in these partnerships [14]. Furthermore, use of ART for safer conception by the partner living with HIV was not widely practiced or recognized in East African serodiscordant partnerships [15].

The objective of this analysis was to examine the associations between fertility desire and fertility intent, condomless sex, detectable HIV viral load, and when these events occur simultaneously creating the potential for HIV transmission in a cohort of Kenyan WLH in discordant relationships. Three main hypotheses were tested. First, we hypothesized that compared to visits when women expressed no fertility desire, there would be increases in condomless sex at visits when women reported fertility desire and fertility intent, motivated by the goal of becoming pregnant. Second, we hypothesized that compared to visits at which women reported no fertility desire, there would be increases in ART adherence and viral suppression at visits with fertility desire and intent, motivated by the desire to minimize transmission risk to sex partners and infants. Third, we hypothesized that when comparing visits when women reported no fertility desire to those with fertility desire and fertility intent, there would be no difference in transmission potential, since increases in condomless sex would be offset by greater ART adherence and viral suppression.

## Materials and Methods

This mixed methods analysis was nested within the *Lifecourse Study*, a group of prospective cohort studies examining the relationship between women’s reproductive life course events, condomless sex, and unsuppressed viral load. This analysis utilized data from a cohort of WLH in discordant couples in Nairobi, Kenya. Women living with HIV were eligible to enroll if they were 18 years or older or emancipated minors. Women were enrolled in the discordant couples cohort if their primary partner was HIV-seronegative, and registered at the Kenyatta National Hospital Discordant Couples Centre with their partner at the time of enrollment.

On enrollment, women completed a face-to-face interview in English or Kiswahili to collect demographic, sexual, behavioral, and partner characteristics. Women were asked if they desired to have more/any children (yes/no) and if they wanted more children, were they currently trying to have children (yes/no). A medical history including HIV history and contraceptive use was collected. Depressive symptoms were assessed using the standardized patient health questionnaire-9 (PHQ-9) [16, 17], and alcohol use was evaluated using the Alcohol Use Disorder Identification Test (AUDIT) [18]. Screening for intimate partner violence (IPV) and partner controlling behaviors was conducted using an adaptation of the World Health Organization Violence Against Women questionnaire [19, 20]. This questionnaire asks about specific acts of physical violence, emotional violence, sexual violence, and controlling behaviors (Supplemental Document I). A complete physical examination was performed including a pelvic examination with sample collection for diagnosis of sexually transmitted infections (STIs) and detection of prostate specific antigen (PSA) in vaginal secretions. This test is a biomarker for condomless sex and detects exposure to semen in the prior 24–48 h [21]. Blood samples were collected for HIV viral load (VL) and CD4 lymphocyte count. Sexually transmitted infections were treated at no cost. Women received ART at the study clinic according to Kenyan National Guidelines.

Participants returned monthly for ART refills and assessment of adherence. Fertility desire and fertility intent were assessed quarterly. Pelvic examinations, genital sample collection, and urine pregnancy tests were also performed quarterly. Screening for depressive symptoms was completed every 6 months, while screening for alcohol use, IPV, and partner controlling behavior were conducted annually.

Case report forms (CRFs) were reviewed daily to ensure that all fields were appropriately completed. Data were entered into an SPSS database. At quarterly intervals, database entries were printed and compared line-by-line to hard copy CRFs to identify and correct any data entry errors.

Women were reimbursed 250 Kenyan shillings (~ $2.50) at each visit to compensate for travel expenses. All women provided written informed consent to take part in this study. Ethical approval was obtained from the Kenyatta National Hospital-University of Nairobi Ethics and Research Committee and the University of Washington Human Subjects Division.

### Laboratory Methods

*Neisseria gonorrhoeae, Chlamydia trachomatis*, and *Trichomonas vaginalis* were diagnosed using nucleic acid amplification testing on vaginal swabs (Aptima; Hologic, San Diego, CA, USA). Prostate specific antigen was detected in genital secretions using a rapid test for the p30 antigen (ABACard, West Hills, CA, USA). HIV ribonucleic acid quantification was performed using the Hologic/Gen-Probe 2nd generation assay, with a lower limit of quantification of 30 copies/mL. Some 100 mL blood samples were diluted six-fold to 600 mLs prior to testing, so viral suppression for this study was defined as ≤ 180 c/mL [22]. Pregnancy testing was performed using urine β-hCG tests. Enumeration of CD4 lymphocyte counts was performed using FACSCount (Becton–Dickinson, Forrest Lakes, NJ, USA).

### Statistical Analyses

The primary exposure was defined as a three-level categorical variable. The category of *no fertility desire* (reference category) was assigned to visits where women reported no desire for more children. The reference category was compared to visits with *fertility desire*, defined as reporting a desire for more children but not currently trying to conceive, and *fertility intent*, defined as currently trying to conceive. Because fertility desire and fertility intent could change over time, and women could contribute to more than one exposure category, data were analyzed at the visit level**.** There were three primary outcomes: detection of PSA, which can detect exposure to semen for 24–48 h after condomless sex [21], detectable viral load, and HIV transmission potential (when PSA is detected at the same visit as a detectable viral load). Secondary outcomes included self-reported condomless sex, abstinence in the past week, three or more sex acts in the past week, two or more partners in the past week, any STI, and pharmacy-verified late refills (defined as 48 h or more past when all medications should have run out). An additional exploratory analysis was performed to compare the primary and secondary outcomes with a binary exposure of visits where women reported *fertility desire* (reference category) versus *fertility intent*. Generalized estimating equations (GEE) with a Poisson family, log link, and independent working correlation structure with robust standard errors were used to calculate risk ratios (RR) and 95% confidence intervals (95% CI). The working correlation structure was used to account for within-person correlation. Robust standard errors were used to account for a situation where an exchangeable correlation may not be appropriate, because visits farther apart may be less alike than visits closer together [23].

For each exposure-outcome pair, multivariable models were constructed that adjusted for potential confounding factors. Age was selected a priori to include in all adjusted analyses based on knowledge of the population and previous analyses suggesting that this is an important potential confounder of the association between fertility desire (and fertility intent) and the study outcomes [22, 24]. In addition, number of previous live births was selected a priori to include in final adjusted models for the association between fertility desire and condomless sex. Additional variables including marital status [5, 6], education (< 8 years of education or ≥ 8 years) [25], IPV in the past year [20, 22, 26–28], controlling behavior by the regular partner [29], alcohol use [28, 30], depression [25, 28], violence by someone other than the regular partner [31], and engaging in sex work [24] were evaluated for inclusion in the final multivariable models using a manual forward step-wise model building approach. Potential confounders that changed the a priori adjusted RR by ≥ 10% were included in the final model.

### Sample Size Calculation

It was estimated that women eligible for or on ART would attend at least 10 visits/year and that a conservative estimate of the incidence of condomless sex is ~ 100/100 person-years (12% of visits). The incidence of late refills was estimated to be ~ 60/100 person-years (10% of visits) [32, 33]. Transmission potential was estimated to occur with a frequency of ~ 20/100 person-years (3% of visits). It was assumed that the fertility desire and fertility intention exposures would be present at 10% of visits. Under these assumptions, a sample size of 120 women in the discordant couples cohort would have > 80% power to detect > 1.95-fold higher odds of outcome events.

### In-depth Interviews

To understand the mechanisms underlying decisions to use condoms and remain adherent to ART in the context of fertility desire and intent, in-depth interviews were conducted with WLH in discordant couples. The women who were interviewed were also participants in the cohort that contributed data to the quantitative analyses. A purposive sample of women who expressed the range of fertility desire and intent (no fertility desire, fertility desire, and fertility intent) were approached at the research clinic and asked if they would be interested in participating in in-depth interviews. Open-ended questions were used to explore participants’ contraceptive choices, condom use, and barriers and facilitators to ART adherence. Seven in-depth interviews were conducted, lasting between 1 and 2 hours. All interviews were conducted by a Kenyan researcher with extensive experience conducting qualitive research related to women’s health (GW). Prior to the interviews, women were informed that the interviewer was part of the study team but had no previous engagement with the participants. Interviews were conducted in English or Kiswahili depending on the women’s preferences and were conducted in a private room at the research clinic and were audio-recorded and transcribed. Interviews in Kiswahili were then translated into English. Authors GW and MCE reviewed all transcripts and analyzed these transcripts together. A rapid assessment was conducted using inductive coding to identify themes around fertility desire, fertility intent, use of condoms, ART adherence, and concerns about HIV transmission [34]. Interpretation of coding was discussed iteratively between the two coders until consensus was reached. Quotes were attributed to participants by age and fertility desire/intention.

## Results

Between July 2013 and March 2017, 160 women were screened, all of whom enrolled. Ultimately, 148 women contributed 3020 visits to this cohort analysis. Women were excluded from the analysis if they had experienced menopause (n = 6), had a hysterectomy (n = 1) or tubal ligation (n = 3), or were pregnant (n = 2). Participants’ median number of follow-up visits was 19 (interquartile range [IQR] 11–28). The percent of expected visits attended per participant ranged from 42 to 115%, with a median of 94% (IQR 87–100%). Four participants (3%) had < 50% of expected visits; 11 (7%) had between 50 and 74% of expected visits; and 133 (90%) had between 75 and 115% of expected visits. The median age of women enrolled in the cohort was 32 years (IQR range 29–37) (Table 1). Most women had eight or more years of education (n = 134, 90%). Seventy-seven percent (*n* = 113) of women were using condoms alone for contraception at enrollment. During the enrollment visit, mild or more severe depressive symptoms were reported by 16% (*n* = 23) of women and 27% (*n* = 40) used alcohol. Controlling behavior by the partner was experienced by 42% (*n* = 61) of women and 30% (n = 44) had experienced intimate partner violence in the past year.

**Table 1:** Baseline characteristics of the study sample

Baseline characteristics of the participants (N = 148)	N (%) or median IQR
Age (n = 147)	32 [29–37]
Highest education (less than 8 years) (n = 147)	15 (10)
Ever married	135 (92)
Casual partner in the last 3 months	14 (9)
Number of previous births	2 [1–3]
Engage in sex work (ever exchanged sex for money or gifts)	0 (0)
Contraceptive use by method (n = 147)	
None, condoms only	113 (77)
DMPA or OCP	21 (14)
IUD, Norplant	13 (9)
Index partner attitude about pregnancy^b^ (n = 139)	
Excited	97 (70)
Neutral	30 (22)
Upset	12 (9)
Depressive symptoms by PHQ-9	
Minimal (0–4)	125 (85)
Mild (5–9)	17 (12)
Mod/Severe (10 or higher)	6 (4)
Alcohol use problems by AUDIT	
Non-drinkers	108 (73)
Minimal (1–6)	32 (22)
Moderate (7–15)	7 (5)
Severe/possible alcohol use disorder (16 or higher)	1 (1)
Ever controlling behaviors by the regular partner^a^ (n = 147)	61 (42)
Any IPV in the past year by the regular partner^a^ (n = 147)	44 (30)
Sexual violence by another person in the past year^c^	4 (3)
Physical violence by another person in the past year^c^	11 (7)
CD4 lymphocyte count ≤ 350 mm3 (n = 146)	47 (32)
Fertility desire categories^d^	
No fertility desire	58 (39)
Fertility desire	58 (39)
Fertility intent	32 (22)

*ART* antiretroviral therapy; *AUDIT* Alcohol Use Disorders Identification Test; *DMPA* depot medroxyprogesterone acetate; *IPV* intimate partner violence; *IQR* interquartile range; *OCP* oral contraceptive pills; *PHQ-9* Patient Health Questionnaire 9

^a^Index partner refers to a woman’s current or most recent regular partner (boyfriend or husband) who was not a client. If she did not have a regular partner, she was asked to refer to her most recent regular partner. All IPV questions refer to acts committed by this partner

^b^Asked of women who were not currently pregnant at that visit

^c^These questions refer to someone other than the regular partner

^d^Fertility desire category definitions: No fertility desire when women reported no desire for more children; Fertility desire when women reported a desire for more children but not currently trying to conceive; Fertility intent when women were currently trying to conceive

### Sexual Behaviors

Prostate specific antigen was detected in vaginal secretions at 11.5% (74/644) of visits when women reported no fertility desire. Detection of PSA was slightly lower at visits with fertility desire (8.9%, 42/470; RR 0.78, 95% CI 0.50–1.20; χ^2^ 1.29), and slightly higher at visits with fertility intent (14.1%, 27/191; RR 1.23, 95% CI 0.76–2.00; χ^2^ 0.70; Table 2). The association of PSA with fertility desire was stronger in analyses adjusted for potential confounding factors including age and number of live births (adjusted risk ratio [aRR] 0.63, 95% CI 0.38–1.03; χ^2^ 3.46), though this association did not reach statistical significance. Visits with fertility intent had a significantly higher risk of PSA detection than visits with fertility desire in both unadjusted (RR 1.58, 95% CI 0.96–2.62; χ^2^ 3.18) and adjusted analyses (aRR 1.73, 95% CI 1.02–2.94; χ^2^ 4.12).

**Table 2:** Unadjusted and adjusted associations between fertility desire, fertility intent, and biological as well as self-reported outcomes related to sexual behavior and antiretroviral use in WLH in serodiscordant partnerships

Exposure	# with outcome/# of visits in category (%)	RR (95% CI), χ^2^ (1 df)	p-value	aRR (95% CI), χ^2^ (1 df)	p-value
PSA detection (n = 1305)					
No fertility desire	74/644 (11.5%)	Referent	–	Referent	–
Fertility desire	42/470 (8.9%)	0.78 (0.50, 1.20), 1.29	0.3	0.63 (0.38, 1.03)^1^, 3.46	0.06
Fertility intent	27/191 (14.1%)	1.23 (0.76, 2.00), 0.70	0.4	1.09 (0.63, 1.89)^1^, 0.09	0.8
Fertility intent vs fertility desire		1.58 (0.96, 2.62), 3.18	0.08	1.73 (1.02, 2.94)^1^, 4.12	0.04
Self-reported condomless sex in the past week (n = 3014)					
No fertility desire	125/1514 (8.3%)	Referent	–	Referent	–
Fertility desire	36/1060 (3.4%)	0.41 (0.20, 0.86), 5.51	0.02	0.31 (0.15, 0.64)^1^, 9.76	0.002
Fertility intent	28/440 (6.4%)	0.77 (0.34, 1.75), 0.39	0.5	0.68 (0.28, 1.66)^1^, 0.74	0.4
Fertility intent vs fertility desire		1.87 (0.94, 3.75), 3.14	0.08	2.21 (1.08, 4.50`)^1^, 4.73	0.03
No sex in the past week (n = 3015)					
No fertility desire	793/1514 (52.4%)	Referent	–	Referent	–
Fertility desire	459/1061 (43.3%)	0.83 (0.68, 1.01), 3.65	0.06	0.89 (0.72, 1.09)^1^, 1.27	0.3
Fertility intent	137/440 (31.1%)	0.59 (0.45, 0.78), 14.33	< 0.001	0.64 (0.48, 0.86)^1^, 8.92	0.003
Fertility intent vs fertility desire		0.72 (0.55, 0.94), 6.02	0.01	0.72 (0.55, 0.95)^1^, 5.46	0.019
≥ 3 sex acts in the past week^b^ (n = 1626)					
No fertility desire	171/721 (23.7%)	Referent	–	Referent	–
Fertility desire	194/602 (32.2%)	1.36 (0.95, 1.94), 2.89	0.09	1.44 (1.00, 2.08)^1^, 3.77	0.05
Fertility intent	90/303 (29.7%)	1.25 (0.81, 1.94), 1.02	0.3	1.21 (0.77, 1.92)^1^, 0.68	0.4
Fertility intent vs fertility desire		0.92 (0.64, 1.32), 0.19	0.7	0.84 (0.58, 1.23)^1^, 0.78	0.4
Any STI (n = 1300)					
No fertility desire	19/642 (2.96%)	Referent	–	Referent	–
Fertility desire	6/471 (1.27%)	0.43 (0.16, 1.19), 2.64	0.1	0.32 (0.11, 0.97)^1^, 4.03	0.05
Fertility intent	2/187 (1.07%)	0.36 (0.05, 2.61), 1.02	0.3	0.26 (0.03, 2.11)^1^, 1.58	0.2
Fertility intent vs fertility desire		0.84 (0.10, 7.15), 0.03	0.9	0.81 (0.10, 6.76)^1^, 0.04	0.8
Late refills (n = 2711)					
No fertility desire	83/1400 (5.9%)	Referent	–	Referent	–
Fertility desire	56/935 (6.0%)	1.01 (0.65, 1.56), 0.00	1.0	0.68 (0.44, 1.03)^3^, 3.31	0.07
Fertility intent	14/376 (3.7%)	0.63 (0.33, 1.18), 2.07	0.2	0.51 (0.29, 0.92)^3^, 4.93	0.03
Fertility intent vs fertility desire		0.62 (0.34, 1.12), 2.50	0.1	0.76 (0.44, 1.32)^3^, 0.95	0.3
Detectable viral load (n = 700)					
No fertility desire	51/341 (15.0%)	Referent	–	Referent	–
Fertility desire	32/257 (12.5%)	0.83 (0.47, 1.48), 0.39	0.5	0.68 (0.43, 1.09)^3^, 2.53	0.112
Fertility intent	17/102 (16.7%)	1.11 (0.48, 2.56), 0.06	0.8	1.00 (0.46, 2.20)^3^, 0.01	1.0
Fertility intent vs fertility desire		1.34 (0.59, 3.02), 0.49	0.5	1.47 (0.66, 3.26)^3^, 0.88	0.3
Transmission potential (n = 696)					
No fertility desire	7/338 (2.1%)	Referent	–	Referent	–
Fertility desire	3/256 (1.2%)	0.57 (0.15, 2.11), 0.72	0.4	0.33 (0.08, 1.36)^4^, 2.37	0.1
Fertility intent	4/102 (3.9%)	1.89 (0.49, 7.37), 0.31	0.4	1.46 (0.38, 5.63)^4^, 0.31	0.6
Fertility intent vs fertility desire		3.35 (0.65, 17.17), 3.11	0.1	4.45 (0.85, 23.32)^4^, 3.11	0.078

*PSA* prostate specific antigen test; *RR* risk ratio; *aRR* adjusted relative risk

^a^The final multivariate models were adjusted for *age* (continuous) and *number of live births* at enrollment (continuous)

^b^Among the 1626 women reporting any sex in the past week

^c^The final multivariate models were adjusted for *age* (continuous) and *education* at enrollment (binary)

^d^The final multivariate models were adjusted for *age* (continuous)

To explore whether differences in modern non-barrier contraceptive use might explain the observation that condomless sex was higher at visits without fertility desire compared to visits with fertility desire, modern non-barrier contraceptive use was compared between visits when women reported no fertility desire and visits when fertility desire was reported. Women reported modern non-barrier contraceptive use at 44% (285/652) of visits when they reported no fertility desire compared to 23% (155/665) of visits when they reported fertility desire (χ^2^ 61.6; p < 0.001).

Overall, self-reported condomless sex was slightly less common than PSA detection. Women reported condomless sex in the past week at 8.3% (125/1514) of visits when there was no fertility desire compared to 3.4% (36/1060) of visits when there was fertility desire, and at 6.4% (28/440) of visits when there was fertility intent. In inferential analyses, compared to visits without fertility desire, at visits with fertility desire women reported substantially less condomless sex (RR 0.41 95% CI 0.20–0.86; χ^2^ 5.51). This association was stronger in the adjusted analysis (aRR 0.31, 95% CI 0.15–0.64; χ^2^ 0.76). There was not a meaningful difference in reported condomless sex between visits with no fertility desire and visits with fertility intent (RR 0.77, 95% 0.34–1.75; χ^2^ 0.39; aRR 0.68, 95% CI 0.28–1.66; χ^2^ 0.74).

In comparison to the U-shaped relationships seen in PSA detection and condomless sex where the lowest prevalences were in women with fertility desire, there were stepwise declines in abstinence at visits when women reported no fertility desire (52.4%; 793/1514), compared to visits with fertility desire (43.3%; 459/1061), and fertility intent (31.1%; 137/440). The GEE results highlight these findings. Compared to visits without fertility desire, abstinence in the past week was less common at visits when women reported fertility desire (RR 0.83 95% CI 0.68–1.01; χ^2^ 3.65; aRR 0.89, 95% CI 0.72–1.09; χ^2^ 1.27). Similarly, when fertility intent was expressed, abstinence was less frequent when compared to visits without fertility desire (RR 0.59, 95% CI 0.45–0.78; χ^2^ 14.33; aRR 0.64, 95% CI 0.48–0.86; χ^2^ 8.92).

Reporting a sex frequency of three or more episodes per week (the median for the cohort) varied when comparing visits without fertility desire (23.7%, 171/721), to visits with fertility desire (32.2%, 194/602), and with fertility intent (29.7%, 90/303). Compared to visits without fertility desire, higher sexual frequency was reported more often at visits with fertility desire (RR 1.36, 95% CI 0.95–1.94; χ^2^ 2.89; aRR 1.44, 95% CI 1.00–2.08; χ^2^ 3.77). In contrast, higher sexual frequency did not differ greatly between visits without fertility desire and visits with fertility intent (RR 1.25, 95% CI 0.81–1.94; χ^2^ 1.02; aRR 1.21, 95% CI 0.77–1.92 χ^2^ 0.68).

Sexually transmitted infections were relatively uncommon in this population but varied in relation to women’s reported fertility desire and fertility intent. The prevalence of STIs was 3.0% (19/642) at visits without fertility desire compared to 1.3% (6/471) of visits with fertility desire, and 1.1% (2/187) of visits with fertility intent. In inferential analyses, compared to visits without fertility desire, the risk of having an STI was significantly lower at visits with fertility desire (RR 0.43, 95% CI 0.16–1.19 χ^2^ 2.64; aRR 0.32 95% CI 0.11–0.97; χ^2^ 4.03). Similarly, compared to visits without fertility desire, STIs were diagnosed less frequently at visits with fertility intent (RR 0.36, 95% CI 0.05–2.61; χ^2^ 1.02; aRR 0.26, 95% CI 0.03–2.11; χ^2^ 1.58), though confidence intervals were wide and this association was not statistically significant.

### Late ART Refills and Detectable HIV Viral Load

Late refills occurred at 5.9% (83/1400) of visits when fertility desire was not expressed, at 6.0% (56/935) of visits with fertility desire, and at 3.7% (14/376) of visits with fertility intent. Compared to visits without fertility desire, the risk of late refills was similar at visits with fertility desire in unadjusted analyses (RR 1.01, 95% CI 0.65–1.56; χ^2^ 0.00). However, after adjustment for age and education, there was a statistical trend suggesting a lower risk of late refills when fertility desire was expressed compared to visits without fertility desire (aRR 0.68, 95% CI 0.44–1.03; χ^2^ 3.31). Similarly, late refills occurred less frequently at visits when women reported fertility intent compared to visits without fertility desire (RR 0.63, 95% CI 0.33–1.18; χ^2^ 2.07; aRR 0.51 95% CI 0.29–0.92; χ^2^ 4.93).

Detectable viral load patterns were somewhat different than late refills. Detectable viral loads occurred at 15.0% (51/341) of visits when women reported no fertility desire, 12.5% (32/257) of visits with fertility desire, and at 16.7% (17/102) of visits with fertility intent. In GEE analyses, compared to visits without fertility desire, the risk of detectable viral loads was lower at visits with fertility desire (RR 0.83, 95% CI 0.47–1.48; χ^2^ 0.39). This association was stronger in adjusted analyses (aRR 0.68, 95% CI 0.43–1.09; χ^2^ 2.53), though the difference was not statistically significant. There was a similar risk of detectable viral loads when comparing visits without fertility desire to visits with fertility intent (RR 1.11, 95% CI 0.48–2.56; χ^2^ 0.06; aRR 1.00, 95% CI 0.46–2.20; χ^2^ 0.01), although this association also was not statistically significant.

### Transmission Potential

Transmission potential was uncommon and followed a U-shaped pattern that was similar to PSA detection. Specifically, a detectable plasma HIV viral load was present at the same time as detection of PSA in vaginal secretions at 2.1% (7/338) of visits without fertility desire, compared to 1.2% (3/256) of visits with fertility desire, and 3.9% (4/102) of visits with fertility intent. In inferential analyses, compared to visits without fertility desire, the risk of transmission potential was lower at visits with fertility desire (RR 0.57, 95% CI 0.15–2.11; χ^2^ 0.72; aRR 0.33, 95% CI 0.08–1.36; χ^2^ 2.37) and higher at visits with fertility intent (RR 1.89, 95% CI 0.49–7.37; χ^2^ 0.31; aRR 1.46, 95% CI 0.38–5.63; χ^2^ 0.31), though confidence intervals were wide and results were nonsignificant for both comparisons.

### In-depth Interviews

**Fig. 1:**
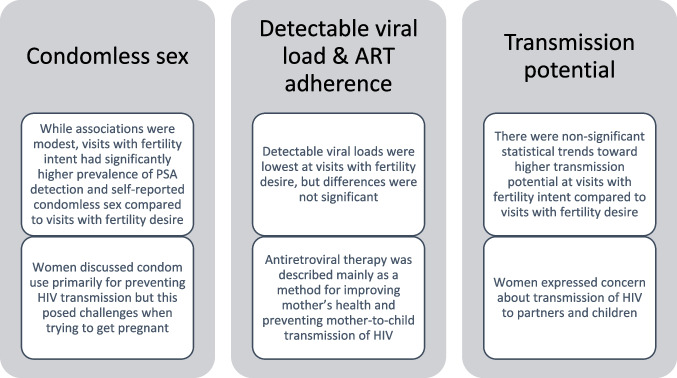
Joint display including summaries of key quantitative and qualitative results

In-depth interviews were conducted with seven cohort participants between September 2014 and October 2014. Themes arose around the importance of children to WLH, condom use to prevent HIV transmission rather than pregnancy prevention, and that antiretroviral therapy was being used to improve the woman’s health and decrease transmission during pregnancy. These results are presented alongside the quantitative results in Fig. 1.

### Importance of Children to WLH in Discordant Partnerships

Themes around community pressure to have children, cultural norms, and a woman’s identity being tied to her children were present in all interviews.

“A woman is not a woman if she doesn’t bear children.” (27 years old, fertility desire)

“A woman cannot be complete without children.” (37 years old, no fertility desire)

These data underscore the fact that fertility and children may be highly important drivers of behavior in this population.

The effect of a woman’s HIV status on fertility desire was mixed. Some women described hesitancy to have more children because of their HIV status and concerns about potential transmission to their baby.

No […] We are not planning to have another one just because with the agony I passed through with the pregnancy of this boy, the uncertainty of the status […] That is the main reason: the uncertainty of the status of the boy and even my status too. Carrying the pregnancy, taking care of the pregnancy with the status of HIV, I cannot do it. It’s really stressful. (42 years old, no fertility desire)

However, in other interviews, women articulated that HIV did not change their views about having children.

It cannot prevent me. The only thing is that I take care of myself and ensure that I follow everything that they have told me, my CD4 is okay, viral load is low so that I can get a baby who is okay at least, so that he can have a good life. (27 years old, fertility desire)

Ultimately, community pressure and individual women’s desire to have children were strong. However, HIV did impact some women’s decision about whether to have additional children.

### Condoms Used Primarily to Prevent HIV Transmission Rather Than Pregnancy

Another common theme was that condoms were often used to prevent transmission to women’s HIV-seronegative partners, rather than explicitly for pregnancy prevention.

“We use a condom because I don’t want to infect him […] About family planning, I get injections...” (42 years old, no fertility desire)

Interviewer: What is the main reason you two use a condom?

*Interviewee: “So that I don’t infect him”* (24 years old, fertility desire).

However, using condoms to prevent transmission of HIV to sexual partners was challenging if women were intending to get pregnant.

“Because he is very keen when it comes to using condoms. He couldn’t accept even if I told him otherwise. So getting pregnant is not that easy.” (24 years old, fertility desire)

Less commonly, women reported using condoms specifically for pregnancy prevention.

“Condoms also help prevent unwanted pregnancies and I don’t want to use ‘drugs’ [contraceptive pills].” (27 years old, fertility desire)

### Antiretroviral Therapy to Improve the Mother’s Health and Prevent HIV Transmission During Pregnancy

A common theme of ART use was women’s desire to improve their own health so they could continue caring for their existing children.

“Looking at my children I thought if I was not to live positively, I would die leaving them young, and my husband is not very supportive towards our family. So, I felt that if I was to die, my children may suffer a lot. So, I decided if I was to raise my children then I needed to adopt that positive attitude, avoid stress, eat right so that I may live like a normal person.” (37 years old, no fertility desire)

“That’s when I was told I will have to take the medication so that I may live and raise my child. That’s when I accepted my status.” (37 years old, no fertility desire)

Antiretroviral therapy was also recognized as a method for decreasing mother-to-child transmission of HIV during pregnancy.

“[…] I asked here and there, and I got to learn that there’s a risk [of HIV being transmitted to your baby], albeit small, because nowadays there are drugs which can be taken to minimize transmission. Also, I learnt that when you are going to give birth, it’s better to be free with your doctors and tell them about your condition so that they can do something to prevent your infected blood from mixing with your baby’s during childbirth.” (27 years old, fertility desire)

Interviewer: What steps were taken to ensure that your child will not be infected with HIV?

Interviewee: “ […]if I took the medicine I was told my child will not get infected. [So] I started taking the medication when I was two months pregnant.” (37 years old, no fertility desire)

Women also discussed the role of ART and decreasing transmission to seronegative partners. However, this benefit of ART was less commonly discussed than the other reasons for ART use.

“My CD4 count used to be 253 now it’s risen to almost 1000, I’m okay. The drugs are okay and they’re helping me so it’s harder to infect him.” (27 years old, fertility desire)

However, women did not always verbalize the link between ART adherence, undetectable viral load, and prevention of sexual transmission of HIV. Even in the setting of viral suppression, there were concerns about HIV transmission if condoms were not being used. This is contrary to the Undetectable = Untransmissable (U = U) messaging that stresses to people living with HIV that an undetectable viral load means that HIV is not transmissible to others.

Interviewer: […] If you use ARTs, can you have unprotected sex, if you have reduced your viral load?

Interviewee: “No, not really, because the virus is still present […] they hide, they are still present.” (42 years old, no fertility desire)

Partners were supportive of women taking ART and were facilitators of adherence.

“He actually reminds me. I usually take the ARV at nine o’ clock. He calls me at eight fifty-nine. Even if he doesn’t have credit, he will flash me. When he flashes and I check my phone, it is nine o’ clock.” (24 years old, fertility desire)

**“**My husband knows, he used to wake me up before midnight he used to put the alarm for me and wake me up and tell me wake up and take your drugs…” (39 years old, no fertility desire)

## Discussion

In this population of WLH in serodiscordant partnerships, children were often viewed as a central element of womanhood. At baseline, two out of five women reported that they would like to have more children in the future, and another one in five was actively trying to become pregnant. Detection of PSA, a biomarker for recent condomless sex, was lowest in women with fertility desire and highest in women with fertility intent, with a significant increase when comparing visits with fertility desire to those with fertility intent. Women expressed a strong reliance on condom use for prevention of sexual HIV transmission but seemed to know less about how ART can also reduce sexual transmission risk.

Other studies have documented similarly complex relationships between fertility desire, fertility intent, and HIV transmission risk in African HIV-serodiscordant couples [35, 36]. For example, a study of South African WLH identified a strong desire to experience parenthood despite a strongly perceived community disapproval associated with HIV and reproduction [35]. Likewise, a study of HIV-serodiscordant couples in Tanzania and South Africa found that fertility desire and fertility intent were common, but partners living with HIV expressed fear of transmitting HIV to their seronegative partners [37]. These competing priorities resulted in a tension between using condoms to prevent HIV transmission to partners and condomless sex to get pregnant.

While current guidance for discordant couples highlights the effectiveness of ART to prevent HIV transmission [38–40], women in this study associated their ART use mainly with improving their health to care for their existing children. Less frequently, women discussed the association between viral load suppression and decreased transmission to partners and infants. Importantly, these interviews were conducted in 2014 before the Undetectable = Untransmissible (U = U) terminology was in use and widely disseminated. A 2013 qualitative study of serodiscordant couples in Kenya showed a similar lack of awareness about the ability of ART to prevent sexual transmission of HIV [41]. This contrasts with a 2018 study that demonstrated high uptake of PrEP and ART for safer conception in serodiscordant couples in Kenya and Uganda [40]. The evolving findings from these studies likely demonstrate the temporal shift in knowledge about ART use to prevent sexual transmission of HIV.

It is important to stress how these findings fit into the larger discussion about safer conception in discordant partnerships and preventing-mother-to-child transmission (PMTCT) programs. These results highlight the fear and stress that women feel about the risks of transmitting HIV to their baby when thinking of future pregnancies. The U = U messaging was not widely disseminated at the time of this study, and U = U is based on preventing sexual transmission of HIV [42]. Safer conception clinics and antenatal clinics could provide clearer messaging about the relationship between viral load and vertical transmission risk, similar to the U = U messaging for sexual transmission, to reduce stigma and empower women with the tools to minimize transmission risk to their infant.

While the observed differences were modest, we noted that visits with fertility desire were associated with the lowest prevalences of both condomless sex and detectable viral load***.*** The observed differences in condomless sex could be explained by differences in modern non-barrier contraceptive use, since visits where women reported no fertility desire were associated with more modern non-barrier contraceptive use than at visits when women reported fertility desire. Given these differences in modern non-barrier contraceptive use, it is possible that condoms are used more commonly for pregnancy prevention when women have fertility desire but are not currently trying to become pregnant. Future research could help to determine whether a similar phenomenon is observed in other populations of serodiscordant couples. Regardless, these results make it clear that for WLH, the transition from desiring more children in the future to actively trying to become pregnant may represent an important reproductive life course event during which additional measures could be helpful in minimizing the risk of sexual HIV transmission.

This study had several strengths. The longitudinal cohort allowed for repeated assessment of exposures and outcomes over time, providing insight into the temporal relationships between variables. The use of biologic measures of condomless sex and viral load suppression avoided the potential for recall and social desirability biases. In addition, combining these biological measures with self-reported sexual behaviors and medication refill data provided a more comprehensive picture of behaviors. Finally, despite the relatively small number of in-depth interviews, these qualitative data provided insight into women’s reasons for condom use and adhering to ART, offering context and illustrating potential mechanisms that help to explain the quantitative findings.

There are also some limitations to note. First, the reproductive plans of WLH may be a sensitive topic due to negative societal perceptions of pregnancy in this population. This social desirability bias might be expected to result in under-reporting of fertility intent, causing bias in the risk ratios for our outcomes that are not easy to predict. This limitation was minimized by having experienced research staff administer the questionnaires in a non-judgmental way that was supportive of all reproductive choices, and in the setting of a discordant couples care center where participants are encouraged to discuss their reproductive plans. Second, PSA detection in vaginal secretions only detects condomless sex during the past 24–48 h. This does not bias comparisons of PSA detection between groups but means that the rate of PSA detection reflects only a fraction of all episodes of condomless sex. Third, this study did not include HIV resistance testing, so it is not possible to differentiate viremia due to current poor adherence from viremia caused by resistance to ART. If fertility desire and fertility intent impact viral load through effects on ART adherence, the relation between these reproductive choices and viral load could be attenuated by the effects of ART resistance. Fourth, the cohort study was conducted between 2013 and 2017 and the interviews were conducted in 2014, prior to widespread dissemination of the U = U messaging. While this does not impact the internal validity of the results, it will be important to consider the changing context when comparing these results to contemporary populations. Finally, because the analysis of qualitative data was conducted after the cohort study was completed, a member check of the qualitative findings with participants was not possible.

## Conclusions

In conclusion, these results suggest that in WLH women in serodiscordant partnerships, the transition from wanting more children in the future to actively trying to conceive is a potential point for intervention to reduce the risk of sexual HIV transmission. Fortunately, overall transmission risk was relatively low. However, supporting WLH by improving their understanding of the role of ART in preventing sexual transmission, performing viral load monitoring with resistance testing if indicated, providing adherence support with evidence-based interventions, and timing condomless sex to the period of peak fertility could help to support safer conception.

## Data Availability

This study was conducted with approval from the Kenyatta National Hospital—University of Nairobi Ethics and Research Committee (KNH-UON ERC), which requires that we release data from Kenyan studies (including de-identified data) only after they have provided their written approval for additional analyses. As such, data for this study will be available from the authors upon request, with written approval for the proposed analysis from the KNH/UON ERC. Their application forms and guidelines can be accessed at https://erc.uonbi.ac.ke/. Please contact the KNH-UON ERC at principal-cae@uonbi.ac.ke.
